# Case of a Punctured Wound of the Abdomen, Accompanied with Injury to the Intestinal Canal, and to Some Blood-Vessel of Considerable Size

**Published:** 1828-09

**Authors:** H. C. Field

**Affiliations:** Member of the Royal College of Surgeons in London.


### WOUND OF THE ABDOMEN.

*Case of a punctured Wound of the Abdomen, accompanied with Injury to the Intestinal Canal, and to some Blood-vessel of considerable size.* Communicated by H. C. Field, m.d. Member of the Royal College of Surgeons in London.

On the evening of the 22d of November, 1824, I was called to see Captain ——, who, I was informed, was about thirty-five years of age, and had, in a fit of irritation, inflicted some serious injury on himself. On entering his room, I found the floor covered with blood, and himself extended on a sofa apparently lifeless. I could not feel any pulse, but immediately gave him a little wine, which he swallowed with difficulty. I soon perceived that blood was flowing in considerable quantity from a wound in the abdomen, which was situated at the left side of, and near to, the umbilicus. At this moment I was assisted in the examination and treatment of the case by my friend Mr. Taggert, member of the Royal College of Surgeons in Ireland. We soon learned that the wound had been inflicted by a large carving knife, which, from the appearance it presented, and from the account we received, as well as from the nature of the wound, we judged had passed into the cavity to the distance of four or five inches, in a direction obliquely backwards, downwards, and to the left side. The blood, which was of a dark colour, flowed freely in one continued stream, and the hemorrhage was so excessive as to excite our apprehensions of immediate dissolution. However, on introducing to the bottom of the wound long dossils of lint, dipped in turpentine, the ends being kept outside the wound, and applying over them graduated compresses and a bandage, we succeeded in a short time in arresting the further flow of blood. A cordial opiate was next administered. A feeble pulse at the wrist could now be felt, but he appeared in a sort of faint; he was bathed in a cold sweat, his extremities lay still and motionless, he vomited at intervals, his urine and feces passed unconsciously to him, and his respiration was slow and laboured: in this state, almost bordering on death, he remained for some hours. About this time the vital powers first showed a disposition to reaction: he expressed in a few broken accents the general uneasiness he felt, and complained of pain and numbness in the lower extremities, particularly in that of the left side. This, with a peculiar restlessness, disturbed him during the remainder of this night, which was passed without any sleep, notwithstanding the anodyne he had taken.

November 23d.—A medical attendant remained with him during the night. There was no recurrence of hemorrhage; he still appears in a state of extreme debility, and feels great nausea, which occasionally increases to hiccough and vomiting; he also complains of numbness and pain extending down the left limb to the foot; slight tenderness about the wound on pressure; pulse 120, feeble and irregular. With a view of relieving the state of the stomach, as well as of allaying the general irritability, the saline mixture was directed every third or fourth hour, in a state of effervescence, with fifteen drops of laudanum in each draught. Fomentations to the abdomen.

Evening.—He still complains of the pain and numbness in the thigh and legs; the tenderness of the abdomen on pressure is augmented; thirst; pulse 100, and feeble; tongue white and furred; skin hot and dry; stomach relieved.

Ordered to continue the effervescing and opiate draughts, at longer intervals. An emollient enema every fourth hour; and Olei Ricini ℥ss. in the morning.

24th.—Has had some quiet and refreshing sleep during the night, and feels himself relieved this morning. Pulse ninety; skin cool; no sickness of stomach. Has had two copious alvine evacuations, each containing a quantity of dark clotted blood. Pain and numbness in the affected limb not so troublesome; tenderness of abdomen on pressure continues.

Ordered, the enemata and fomentations to be repeated; also the effervescing mixture, with small doses of Tartar Emetic.

25th.—Has had several copious evacuations, each containing clotted blood. Fever much less; pulse eighty-six; tongue still foul; pain and numbness in the extremity still distresses him; there is little or no pain about the wound. This day the dressings were removed for the first time, when a quantity of feculent matter, mixed with blood and pus, was discharged. The wound was then dressed superficially; an oil draught directed; and absolute rest enjoined.

26th.—Has had some refreshing sleep, and is much better this morning. Passed several evacuations: the quantity of blood in each has considerably diminished.

27th.—Continues to improve. The evacuations are free from blood. He now complains of occasional startings in the left limb, in addition to the pain and numbness, which still continue, though in a less degree. The wound discharges healthy pus, unmixed with feculent matter. He is free from fever.

I think it unnecessary to delay my readers by detailing the daily reports. He continued to improve daily: the wound gradually healed; the peculiar sensations of numbness, pain, and tingling in the left leg and thigh, however, continued, and were occasionally very distressing, although, on the whole, less severe and frequent in their attack. On the 8th of December, he attempted to stand, but the left leg was so weak as to yield beneath his weight.

January 2d.—Since the last report, his health and strength have considerably improved. For some time the left extremity was nearly paralytic, though frequently attacked with paroxysms of pain and numbness; which latter he describes as more troublesome and distressing than the former. These paroxysms, however, are now so slight and so few, that every hope may be entertained of his perfect recovery at no very distant period.

I cannot close this brief statement without directing the attention more particularly to some of the circumstances of this interesting case, which I conceive are calculated to excite our astonishment, as well as our admiration at the extraordinary resources of nature, whereby she is enabled, under the most unfavorable circumstances, to preserve the life of an individual.

In this case we have evidence not only of the peritoneum, but also of the intestinal canal, being opened. I should presume, from the feculent quality of the discharge, that some part of the colon was injured. What blood-vessel was wounded, cannot with any certainty be affirmed; but most probably some large branch of the vena portæ, such as the inferior mesenteric, or one of the colic veins. I conceive there can be no doubt, from the peculiar sensations complained of in the lower extremity, that some branches of the lumbar plexus of nerves must have been divided, or otherwise injured, at the time of the accident.

It appears a strange anomaly in the history of wounds of the abdomen, that so little peritoneal inflammation was excited in this case, notwithstanding the effusion of blood and feculent matter. May we not in some degree account for the fortunate absence of this so common a result in general, either by the copious hemorrhage which immediately followed reducing the system to so low a condition as to retard reaction in the circulatory organs, and so to oppose the attack of local inflammation or general fever? or a coagulum of blood may have so formed around the wounded vessel, as that further bleeding and effusion into the cavity were restrained until the adhesive inflammation set in, by means of which a permanent recovery was effected in a surprisingly short time; for we find him in less than a fortnight free from every unpleasant symptom, excepting the pain and numbness in the lower extremity, which may be referred to the injury of some of the nervous filaments of the limb.

